# Feasibility, reliability and validity of the health-related quality of life instrument Child Health Utility 9D (CHU9D) among school-aged children and adolescents in Sweden

**DOI:** 10.1186/s12955-021-01830-9

**Published:** 2021-08-03

**Authors:** Kristina Lindvall, Masoud Vaezghasemi, Inna Feldman, Anneli Ivarsson, Katherine J. Stevens, Solveig Petersen

**Affiliations:** 1 Department of Epidemiology and Global Health, Umeå University, 901 87 Umeå, Sweden; 2 Department of Public Health and Caring Science, Uppsala University, Uppsala, Sweden; 3 School of Health and Related Research, The University of Sheffield, Sheffield, UK

**Keywords:** Child, Adolescent, CHU9D, Health related quality of life, HRQoL, Psychometrics, Sweden

## Abstract

**Background:**

This study was conducted in a general population of schoolchildren in Sweden, with the aim to assess the psychometric properties of a generic preference-based health related quality of life (HRQoL) instrument, the Swedish Child Health Utility 9D (CHU9D), among schoolchildren aged 7–15 years, and in subgroups aged 7–9, 10–12 and 13–15 years.

**Methods:**

In total, 486 school aged children, aged 7–15 years, completed a questionnaire including the CHU9D, the Pediatric quality of life inventory 4.0 (PedsQL), KIDSCREEN-10, questions on general health, long-term illness, and sociodemographic characteristics. Psychometric testing was undertaken of feasibility, internal consistency reliability, test–retest reliability, construct validity, factorial validity, concurrent validity, convergent validity and divergent validity.

**Results:**

The CHU9D evidenced very few missing values, minimal ceiling, and no floor effects. The instrument achieved satisfactory internal consistency (Cronbach’s Alfa > 0.7) and strong test–retest reliability (r > 0.6). Confirmatory factor analyses supported the proposed one-factor structure of the CHU9D. For child algorithm, RMSEA = 0.05, CFI = 0.95, TLI = 0.94, and SRMR = 0.04. For adult algorithm RMSEA = 0.04, CFI = 0.96, TLI = 0.95, and SRMR = 0.04. The CHU9D utility value correlated moderately or strongly with KIDSCREEN-10 and PedsQL total scores (r > 0.5–0.7). The CHU9D discriminated as anticipated on health and on three of five sociodemographic characteristics (sex, age, and custody arrangement, but not socioeconomic status and ethnic origin).

**Conclusions:**

This study provides evidence that the Swedish CHU9D is a feasible, reliable and valid measure of preference-based HRQoL in children. The study furthermore suggests that the CHU9D is appropriate for use among children 7–15 years of age in the general population, as well as among subgroups aged 7– 9, 10–12 and 13–15 years.

## Introduction

Economic evaluations, including cost-utility analysis (CUA), have a central role in healthcare decision-making [1]. CUA is typically expressed as the incremental cost of interventions per quality adjusted life years (QALYs), a common outcome unit that enables comparisons across clinical areas. The QALYs can be calculated by multiplying the duration of time spent in a health state by the health related quality of life (HRQoL) utility weight associated with that health state, using the area under the curve (AUC) method [1]. HRQoL can be captured by multi-attribute utility (MAU) instruments addressing key generic health domains.

Existing Pediatric MAU instruments are mostly adapted from adult instruments or developed from the perspective and preferences of adults [2–4]. Such adult-based HRQoL measures may be cognitively challenging for younger populations [5]. They may also capture health aspects less pertinent to pediatric populations, while failing to tap into others of particular importance to children’s HRQoL, and adult preferences for health states may differ from child preferences [6]. To overcome some of these problems, a MAU instrument, the Child Health Utility 9D (CHU9D), was developed specifically with and for children [7–9], and with scoring algorithms obtained from a parent [10] as well as a child population [11].

The Original English version of the CHU9D has demonstrated sound psychometric properties in 7 to 11-year-olds in the UK [8] and in 11 to 17-year-olds in Australia [12–14]. Linguistic and cognitive skills, however, vary over the course of childhood and the ability to understand and respond to a questionnaire may differ between children in different ages. Therefore, acceptable psychometric properties should be assured, also in narrower age strata. Furthermore, to enable valid and reliable CUA in pediatric populations more widely, the CHU9D are being translated into other languages [15]. However, HRQoL is context dependent [16]. Therefore, psychometric properties of translated instruments should be assured in their specific cultural contexts [17].

The CHU9D has been translated into Swedish. The current study is the first to investigate the psychometric properties of the Swedish CHU9D.

This study aimed to investigate the feasibility, reliability, and construct validity of the CHU9D among school-aged children attending grades 1–9 in Sweden (ages 7–15 years), and in subgroups attending grades 1–3, 4–6 and 7–9 (ages 7–9, 10–12 and 13–15 years).

## Methods

### Study population

The study utilized a convenience sample of children attending grades 1–9 (ages 7–15 years) in four elementary schools in a city in Northern Sweden with around 89,000 inhabitants. Around 9000 of these are 7–15 years old. A minimum of 50 children were included from each grade (1–9), resulting in a general population sample of at least 150 children from each of the three elementary school stages: grades 1–3; 4–6; and 7–9 (ages 7–9, 10–12 and 13–15 years). The one exclusion criteria used was (children) not being fluent in Swedish.

### Data collection procedure

Headmasters and class-teachers were informed about the study. Following their consent, children were informed about the study in class and parents received written information. Informed consent to participate was sought from the child. In children below 15 years, also parental consent was requested. After obtained consent, children attending school the day of the survey, filled in a questionnaire in school, assisted by a specially trained research assistant (school nurse). Approximately 59% of the approached students participated in the study. Parents of children in grades 1–3 filled in a short questionnaire at home. In the younger age groups (grades 1–3), the research assistant read the questions and response alternatives aloud, one by one. In each grade, approximately half of the participating schoolchildren filled in the CHU9D again, 7–15 days after the first assessment. Whole classes were randomly selected for the second assessment. Teachers facilitated the process, and were present, but not actively involved in the data collection.

### Measures

The children answered a questionnaire including three measure of HRQoL, the CHU9D, KIDSCREEN-10 [18, 19] and the Pediatric quality of life inventory 4.0 generic core scale (PedsQL) [20, 21], along with measures of health and socio-demographic background. Given the lower cognitive ability of the younger children, some questions were omitted from the child questionnaire in grade 1, and in grades 1–3, some background questions were parent-reported.

#### CHU9D

The preference-based CHU9D measures HRQoL by nine-dimensions: worried, sad, pain, tiredness, annoyed, schoolwork/homework, sleep, daily routines, and ability to join in activities. Recall time is today or last night, and each dimension has five severity levels [7, 22]. Reponses were converted to utilities (on a scale from 0 implying dead to 1 implying perfect health) using both available scoring algorithms: the child-generated Australian algorithm [11] and the adult-generated UK algorithm [10], onwards named child- and adult algorithms.

#### KIDSCREEN-10

KIDSCREEN-10 addresses 8–18-year-old children and captures overall non-preference based HRQoL based upon 10 underlying dimensions: feeling fit and well, full of energy, sad, lonely, and having enough time for one-self, being able to do what you want at spare-time, being treated fairly by parent(s), having fun with friends, doing well in school, and being able to pay attention (in school) [18]. Recall time is one week and items are answered on a 5-point scale from 1 (not at all or never) to 4 (extremely or always). Item scores were recoded into higher values equalling better HRQoL. Then, HRQoL sum scores were calculated and given Rasch-person-parameters (PP), which were further transformed into values with a mean of 50 and a standard deviation of approximately 10 [19]. KIDSCREEN-10 has demonstrated acceptable reliability, construct and criterion validity [18]. This instrument was only filled in by children in grades 2–9.

#### PedsQL

The non-preference based PedsQL has 23 age-specific items that capture 4–18-year-old children’s overall HRQoL, as well as the underlying psychosocial (15 items) and physical (8 items) dimensions [21]. The psychosocial dimension has three sub-dimensions: emotional, social and preschool/school functioning and wellbeing (5 items each). Recall time is one month and items are scored on a 5-point scale between 0 (never a problem) and 4 (almost always a problem). Scores were reversed and linearly transformed to a 0–100 scale with higher scores representing higher HRQoL. Mean values were computed for each dimension. The instrument has demonstrated acceptable psychometric properties in numerous countries, including Sweden [23].

#### Health

Self-reported general health was assessed by the question “How do you feel in general”. The question has five response alternatives, which were merged into three categories: not good/fair (named not good onwards), good, and very good/excellent (named very good onwards). This question was only assessed in grades 2–9.

Long-term illness/disability was studied by asking about the presence of seven specified pediatric chronic or long-term health problems (eczema, asthma, allergies, depression, epilepsy, ADHD, diabetes) and one alternative named “others”. In grades 1–3, this information was obtained from the parents and in grades 4–9 from the child. Children with at least one chronic or long-term health problem were classified as having a long-term illness/disability.

#### Socio-demographic variables

Sex, grade, children’s and parent’s country of birth, and custody arrangement were measured by specific questions. In grades 1–3, parents provided information about country of birth.

Socio-economic status was measured by the Family Affluence Scale (FAS) [24]. FAS assesses self-reported own bedroom, dishwasher at home, number of: family cars, holidays abroad the past 12 months, bath rooms at home and computers at home. A sum score was generated ranging from to 0–13 points, with higher scores indicating higher affluence. The FAS index has shown acceptable cross-cultural reliability and criterion validity in a study of eight European countries, including Denmark and Norway [24].

#### Major life events

To assure equivalence of CHU9D scores at the two assessments used for the test–retest analyses (see statistical analysis), children were asked “Has anything out of the ordinary happened that makes you feel better or worse today”. The response alternatives were no vs yes, please describe. The answers were independently reviewed by two of the authors, (KL and MV) to determine if any child needed to be excluded out of the test–retest analysis due to having a major life event either at the first or second time of participation. If there were any uncertainties, a third author (SP) was consulted.

### Statistical analysis

Differences in CHU9D utility scores were estimated using multivariate tests of means for the one-sample test when applying the child and the adult algorithms, respectively.

*Feasibility* was examined by estimating floor and ceiling effects and the proportion of missing values. The Cronbach’s coefficient α was used to evaluate *internal consistency reliability*, with values ≥ 0.7 considered acceptable for group and ≥ 0.9 for individual comparisons [25]. Furthermore, utility scores from the first and second data collection were compared (*test–retest reliability*) using Intraclass-, Canonical- and Spearman’s correlations for scale comparisons, along with Spearman correlations and Weighted Kappa statistics for dimension comparisons. Correlations of 0.00–0.19 were considered very weak, 0.20–0.39 weak, 0.4–0.59 moderate, 0.60–0.79 strong and 0.80–1.00 very strong [26]. Kappa values below 0.00 were considered to signal poor agreement, 0–0.20 slight, 0.21–0.40 fair, 0.41–0.60 moderate, 0.61–0.80 substantial, and 0.81–1.00 almost perfect agreement [27].

*Construct validity* was assessed through confirmatory factor analysis testing the hypothesized one-factor structure of the CHU9D (*factorial validity*). The following model fit indices and cut-offs were used to confirm model adequacy: comparative fit index (CFI) and Tucker-Lewis index (TLI) values ≥ 0.90 (acceptable fit) or ≥ 0.95 (excellent fit) [28], root mean square error of approximation (RMSEA) values ≤ 0.08 (acceptable fit) or ≤ 0.05 (good fit) [29], and standardized root mean square residual (SRMR) values ≤ 0.08 (acceptable fit) [30]. *Construct validity* was also explored by comparing the total CHU9D utility scores to the total KIDSCREEN-10 and the PedsQL scores, using Spearman’s correlations *(concurrent validity)*. Spearman’s correlations were furthermore used to test whether the conceptually alike dimensions of the CHU9D and the other HRQoL instruments (see Table 1) were correlated (*convergent validity*) and difference between correlations of conceptually alike and dislike dimensions were explored (*divergent validity*).

**Table 1:** Conceptually alike dimensions between the CHU9D and the instruments KID-SCREEN-10 and PedSQL

Instruments		
CHU9D (Dimensions)	KID-SCREEN-10 (Dimensions)	PedsQL (Dimensions)
Worried	–	Emotional functioning
Sad	Felt sad	Emotional functioning
Pain	**–**	Physical functioning
Tired	Felt full of energy	Physical functioning, emotional functioning
Annoyed	–	Emotional functioning
Schoolwork	Got on well in school, able to pay attention (in school)	School functioning
Sleep	–	Emotional functioning
Daily routine	–	Physical functioning
Able to join in activities	Able to do things	Physical functioning, able to join in activities

Finally, *construct validity* was assessed by the *known-groups method,* i.e. by comparing CHU9D utility scores depending on sex, school stages (grades 1–3, 4–6¸7–9), parental country of birth (Sweden: none-one-both parents) custody arrangement (living with both parents-or not), family affluence (FAS: < 25 percentile, 25–75 percentile, > 75 percentile), general health status (not good-good-very good), having a long-term illness or disability (yes–no). Differences between groups were estimated using Mann–Whitney U-test and Kruskal–Wallis test. Lower utility scores were anticipated in girls (although no sex-differences expected in early pre-adolescence) and older age groups, and in those with a foreign background, not living with both parents, having lower family affluence, not holding a good general health, or having a long-term illness or disability [12, 31–33].

The analyses were performed for grades 1–9 in total, and separately for each of the three school stages studied, using Stata version 16.1 (Stata Corp LP, College Station, Texas, USA). Relationships mentioned in the result section are significant at the 95% level (*p* < 0.05).

### Power

The suggested minimum sample size levels for feasibility and reliability tests were n = 50, and in factor analysis 4–10 persons per variable items or at least n = 100 [34]. There is also a rule of thumb stating that validations of questionnaires should include at least 5–10 persons per item, here equivalent to a sample of n = 45–90 [35]. Thus, for the current analyses, the sample size of minimum 50 children at each grade allows for at least school-stage level analysis (n at least 150).

## Results

### Descriptive characteristics

Data was collected from 486 children. Of these, 473 (97%) answered all CHU9D questions and thus, were included in the analysis. These participants were evenly distributed between the three studied school stages, but included slightly less girls than boys (Table 2). The great majority (92%) were born in Sweden, had parents who were both born in Sweden (77%), and lived with both parents (81%). Affluence ranged between 2 and 13 on the Family Affluence Scale, with only two children scoring below 6 points (not seen in the table). Two thirds of the children reported having very good general health and about half reported having a long-term illness/disability, mainly allergy, asthma, and eczema (not seen in the table). HRQoL, as expressed by mean CHU9D utility scores, were 0.74 (SD ± 0.21) when using the child algorithm and 0.85 (SD ± 0.11) when using the adult algorithm (*p* < 0.001). Measured by KIDSCREEN-10 and PedsQL, the corresponding numbers were 41.17 (SD ± 6.10) and 82.12 (SD ± 12.94), respectively.

**Table 2:** Descriptive characteristics of the participants

Characteristics	Total n (%)	School stages		
		Grades 1–3 n (%)	Grades 4–6 n (%)	Grades 7–9 n (%)
Sample	473 (100)	168 (35)	146 (31)	159 (34)
Sex				
Girls	249 (54)	90 (54)	75 (53)	84 (54)
Boys	216 (46)	78 (46)	66 (47)	72 (46)
Children’s country of birth				
Not Sweden	37 (8)	15 (9)	12 (8)	10 (6)
Sweden	436 (92)	153 (91)	134 (92)	149 (94)
Parents’ country of birth				
None Sweden	61 (13)	26 (15)	20 (14)	15 (9)
One Sweden	49 (10)	20 (12)	20 (14)	9 (6)
Both Sweden	363 (77)	122 (73)	106 (72)	135 (85)
Custody arrangement				
Not living with both parents	86 (19)	19 (13)	33 (23)	34 (22)
Living with both parents	366 (81)	130 (87)	112 (77)	124 (78)
Family affluence^1^				
Low (< 25percentile)	69 (16)	24 (18)	23 (16)	22 (14)
Middle (25-75percentile)	288 (66)	86 (62)	95 (67)	107 (69)
High (> 75percentile)	79 (18)	28 (20)	24 (17)	27 (17)
General health^2^				
Not good	64 (15)	14 (12)	16 (11)	34 (22)
Good	78 (19)	16 (14)	26 (19)	36 (23)
Very good	270 (66)	86 (74)	99 (70)	85 (55)
Long-term illness/disability				
Yes	218 (47)	59 (36)	70 (49)	89 (58)
No	243 (53)	107 (64)	72 (51)	64 (42)

	Mean (SD) Median (IQR)	Mean (SD) Median (IQR)	Mean (SD) Median (IQR)	Mean (SD) Median (IQR)
Health related quality of life				
KIDSCREEN-10^3^	41.17 (6.10) 42.00 (38.00–46.00)	41.91 (5.44) 42.00 (38.00–46.00)	42.27 (5.89) 43.00 (39.00–47.00)	39.59 (6.45) 40.00 (36.00–45.00)
CHU9D (child algorithm)^3^	0.74 (0.21) 0.79 (0.59–0.91)	0.80 (0.18) 0.86 (0.70–0.95)	0.75 (0.20) 0.79 (0.62–0.89)	0.65 (0.23) 0.66 (0.49–0.85)
CHU9D (adult algorithm)^3^	0.85 (0.11) 0.87 (0.78–0.93)	0.88 (0.10) 0.91 (0.83–0.95)	0.86 (0.10) 0.88 (0.80–0.93)	0.81 (0.11) 0.82 (0.75–0.90)
PedsQL^3^				
Total score	82.12 (12.94) 84.78 (75.00–92.39)	81.21 (13.74) 83.70 (72.83–91.30)	85.35 (10.57) 88.04 (79.55–94.57)	80.10 (13.57) 81.52 (71.74–89.13)
Physical health	84.85 (13.52) 87.50 (78.12–93.75)	83.62 (14.86) 87.50 (75.00–96.87)	87.42 (11.20) 90.63 (81.25–96.88)	83.76 (13.73) 87.50 (78.13–93.75)
Psychosocial health	80.64 (14.33) 83.33 (73.33–91.67)	79.94 (14.99) 81.67 (71.67–91.67)	84.21 (11.82) 86.67 (76.67–93.33)	78.09 (15.13) 80.00 (71.67–90.00)
Emotional functioning	75.66 (19.03) 80.00 (65.00–90.00)	76.94 (17.54) 80.00 (65.00–90.00)	78.24 (18.32) 80.00 (65.00–90.00)	71.93 (20.66) 75.00 (60.00–90.00)
Social functioning	88.06 (14.74) 95.00 (80.00–100.0)	83.60 (17.33) 87.50 (75.00–95.00)	92.10 (10.17) 95.00 (85.00–100.0)	89.03 (14.16) 95.00 (85.00–100.0)
School functioning	78.31 (16.46) 80.00 (70.00–90.00)	79.34 (15.95) 80.00 (70.00–90.00)	82.45 (13.44) 85.00 (75.00–90.00)	73.44 (18.27) 75.00 (65.00–85.00)

*IQR* inter quartile range, *SD* standard deviation

^1^Low (< 25 percentile) = 2–7 points, Middle (25–75 percentile) = 8–10 points; High (> 75 percentile) = scores 11–13 points on the 0–13-point Family Affluence Scale

^2^Information on general health was only collected in grades 2–9

^3^Higher score means higher health related quality of life

### Feasibility

No child reported the worst possible health state for all nine dimensions (no floor effect), while 8.5% reported the best possible health state, i.e. no problems, for all dimensions (ceiling effect) (Table 3). Stratified analyses revealed varying ceiling effects in the three school stages, i.e. approximately 12% in grades 1–3; 8% in grades 4–6 and 5% in grades 7–9.

**Table 3:** Floor and ceiling effects for the CHU9D among school children in grades 1–9 and stratified by school stages

	All (n = 473)		School stages					
			Grades 1–3 (n = 168)		Grades 4–6 (n = 146)		Grades 7–9 (n = 159)	
Dimensions	Floor^1^ (%)	Ceiling^2^ (%)	Floor^1^ (%)	Ceiling^2^ (%)	Floor^1^ (%)	Ceiling^2^ (%)	Floor^1^ (%)	Ceiling^2^ (%)
Total score	0	8.5	0	11.9	0	8.2	0	5.0
Worried	0.8	73.4	0	83.3	1.4	77.4	1.3	59.1
Sad	1.3	76.5	0	84.5	2.0	75.3	1.9	69.2
Pain	1.7	58.8	1.2	64.9	3.4	53.4	0.6	57.2
Tired	9.3	21.1	7.1	28.0	4.8	23.3	15.7	11.9
Annoyed	0.6	67.6	0	80.9	0.7	69.9	1.3	51.6
Schoolwork	0.2	58.3	0.6	74.4	0	67.8	0	32.7
Sleep	1.7	55.8	2.4	59.5	1.4	58.9	1.3	49.1
Daily routine	0.4	73.4	1.2	76.8	0	71.9	0	71.1
Activities	1.3	62.6	1.2	67.9	2.0	67.8	0.6	52.2

^1^Share of respondents (%) reporting the worst possible health state (highest level of severity)

^2^Share of respondents (%) reporting no problems

Studying each of the nine dimensions separately, floor effects were generally below 2%, and with a few exceptions, this was true across all three school stages. The “tiredness” dimension, however, revealed an overall floor effect of 9%, varying from 5% in grades 4–6 to 16% in grades 7–9. Ceiling effects, on the other hand, overall ranged from 21% (tiredness) to 77% (sadness) and the general pattern of lower ceiling effects by higher school stage was seen for each of the nine dimensions.

### Test–retest reliability and internal consistency

In total, 255 children filled in the CHU9D twice. Of these, 13 children were excluded from the test–retest analyses, 11 because of a major event happening between rating occasions and 2 children had missing values in several dimensions at the second assessment. Thus, 242 children were included in the test–retest analyses, 73 from grades 1–3, 81 from grades 4–6 and 88 from grades 7–9.

At scale level, Canonical, Spearman’s and Interclass correlations all showed strong (> 0.7) or very strong (> 0.8) correlations between the two occasions (Table 4). Similar results were seen for each of the three school stages studied. In addition, all analysis of internal consistency revealed Cronbach alpha values above 0.7. Thus, overall, and at each of the studied school stages, the CHU9D scale met the reliability criteria for group level comparisons.

**Table 4:** Test–retest and internal consistency reliability of the CHU9D at scale level, among school children in grades 1–9, and stratified by school stages (n = 242)

	Test–retest			Internal consistency
	Canonical correlation	Spearman correlation	ICC	Cronbach’s alpha
All	0.81	0.72	0.75	0.79
School stages				
Grades 1–3	0.87	0.74	0.76	0.72
Grades 4–6	0.75	0.62	0.61	0.74
Grades 7–9	0.87	0.75	0.79	0.81

*ICC* intraclass correlation coefficients

When comparing the individual dimensions-scores from the test–retest occasions, one by one, correlations were moderate or strong (0.41–0.71), while kappa-agreement were moderate or close to moderate for 6 dimensions (0.39–0.54) and fair for 2 dimensions (“pain” 0.32 and “annoyed” 0.35) (Table 5). These results were to some extent similar for the separate school stages, but at each of the three school stages, there were cases of weak or non-significant correlations and fair or non-significant kappa-agreements. The specific dimensions holding these weaker test–retest results varied between school stages.

**Table 5:** Test–retest reliability of the CHU9D dimensions, among school children in grades 1–9, and stratified by school stages (n = 242)

Dimensions	Spearman correlation				Coefficient (weighted Kappa)			
	All	School stages			All	School stages		
		Grades 1–3	Grades 4–6	Grades 7–9		Grades 1–3	Grades 4–6	Grades 7–9
Worried	0.49	0.44	0.33	0.49	0.39	0.41	0.27	0.38
Sad	0.57	0.46	0.42	0.70	0.49	0.37	0.32	0.62
Pain	0.42	0.50	0.34	0.46	0.32	0.40	0.25	0.34
Tired	0.56	0.74	0.54	0.36	0.47	0.61	0.43	0.32
Annoyed	0.43	0.24	0.54	0.36	0.35	0.17	0.37	0.32
Schoolwork	0.63	0.52	0.47	0.57	0.54	0.43	0.46	0.47
Sleep	0.48	0.44	0.48	0.51	0.42	0.34	0.42	0.45
Daily routine	0.41	0.33	0.23	0.61	0.39	0.33	0.26	0.54
Activities	0.50	0.41	0.41	0.63	0.40	0.32	0.38	0.45

All correlations and agreements were all statistically significant (*p* < 0.05)

### Construct validity

#### Factorial validity

Confirmatory factor analyses showed that all dimensions loaded significantly on the latent factor and the loadings were all above 0.4, except for abilities to join in activities: 0.32 (child algorithm). Furthermore, as seen in Table 6, the CFI, TLI, SRMR and RMSEA values met the criteria for acceptable or excellent fit for the studied single factor model, i.e. supporting that the nine dimensions of the CHU9D measures a single latent construct. Acceptable model fit was demonstrated when using both child- and adult algorithms, and it was seen in the whole sample (grades 1–9: CFI and TLI ≥ 0.94; RMSEA and SRMR ≤ 0.05), but also in two of the three school stages separately (grades 4–6 and 7–9: CFI and TLI ≥ 0.92; RMSEA and SRMR ≤ 0.06). In grades 1–3, there was weaker support for the model. Modification indices suggested high correlation in the dimensions “worried” and “sad”, which is theoretically plausible. Model fit improved for grades 1–3 after allowing the correlation between these two dimensions in the adjusted model (CFI and TLI 0.83–0.89; RMSEA and SRMR ≤ 0.07).

**Table 6:** Model fit in confirmatory factor analyses testing the CHU9D one-factor structure among school children in grades 1–9 and stratified by school stages (child and adult algorithms) (n = 473)

	RMSEA	CFI	TLI	SRMR
CHU9D (child algorithm)				
All	0.05	0.95	0.94	0.04
School stages				
Grades 1–3	0.09	0.85	0.80	0.07
Grades 1–3, adjusted^1^	0.07	0.89	0.85	0.06
Grades 4–6	0.00	1.00	1.06	0.04
Grades 7–9	0.06	0.94	0.92	0.05
CHU9D (adult algorithm)				
All	0.04	0.96	0.95	0.04
School stages				
Grades 1–3	0.08	0.82	0.76	0.06
Grades 1–3, adjusted^1^	0.07	0.88	0.83	0.06
Grades 4–6	0.00	1.00	1.08	0.04
Grades 7–9	0.03	0.98	0.98	0.05

*CFI* comparative fit index, *RMSEA* root mean square error of approximation, *SRMR* standardized root mean square residual, *TLI* Tucker–Lewis index

CFI and TLI ≥ 0.90 considered acceptable and ≥ 0.95 excellent model fit

RMSEA ≤ 0.8 considered acceptable and ≤ 0.5 good model fit

SRMR ≤ 0.8 considered acceptable model fit

^1^Correlations between dimensions “worried” and “sad” allowed as suggested by the modification indices

### Concurrent validity

Strong correlations were found between the total score of CHU9D (child/adult algorithm) and the total scores of KIDSCREEN (0.61/0.62) and PedsQL, (0.62/0.61). Stratified analyses showed that, in the two older school stages, the CHU9D total scores correlated strongly with the KIDSCREN-10 and PedsQL scores (*r* > 0.6 and 0.7, respectively). In grades 1–3, these correlations were moderate with *r* just above 0.5 (both KIDSCREEN and PedsQL).

### Convergent and divergent validity

The individual nine CHU9D dimensions generally demonstrated moderate (*r* > 0.4), or close to moderate, relationships to the conceptually alike KIDSCREEN-10 and PedsQL dimensions (Table 7). Also, there were a pattern of stronger correlation between alike dimensions as compared to dislike dimensions. One exception was that, unexpectedly, the CHU9D dimension capturing ability to do daily routines had a slightly closer relationship to the PedsQL dimensions emotional and school functioning (*r* 0.38 and 0.36, respectively) than to the physical functioning dimension (*r* 0.31). Another exception was that the dimension on abilities to join in activities showed the strongest relationship with the KIDSCREEN-10 dimension “fit and well” (*r* 0.33). and not the expected dimension regarding ability to do desirable spare time activities (“able to do things” *r* 0.26). In each of the three separate school stages, the results overall follow the same pattern as describes above, although correlations seem to be slightly weaker among the youngest children.

**Table 7:** Correlations between CHU9D dimension scores and dimension scores in KIDSCREEN-10 and PedsQL, among children (n = 473)

	**CHU9D dimensions**								
	**Worried**	**Sad**	**Pain**	**Tired**	**Annoyed**	**School-work**	**Sleep**	**Daily routine**	**Able to join in activities**
KIDSCEEN-10^1^									
Fit/well	− 0.29	− 0.34	− 0.26	− 0.35	− 0.34	− 0.31	− 0.33	− 0.30	− 0.33
Full of energy	− 0.28	− 0.29	− 0.21	− **0.44**	− 0.30	− 0.40	− 0.26	− 0.27	− 0.26
Sad	0.31	**0.48**	0.25	0.26	0.30	0.36	0.31	0.27	0.28
Lonely	0.22	0.45	0.25	0.20	0.27	0.26	0.27	0.28	0.25
Enough own time	− 0.16	− 0.14	− 0.15	− 0.13	− 0.07^3^	− 0.25	− 0.20	− 0.23	− 0.17
Able to do things	− 0.20	− 0.25	− 0.15	− 0.28	− 0.18	− 0.30	− 0.25	− 0.30	− **0.25**
Parents	− 0.10	− 0.16	− 0.10	− 0.11	− 0.11	− 0.16	− 0.19	− 0.19	− 0.20
Friends	− 0.24	− 0.30	− 0.12	− 0.15	− 0.24	− 0.24	− 0.13	− 0.24	− 0.27
Got on well in school	− 0.31	− 0.32	− 0.15	− 0.27	− 0.25	− **0.48**	− 0.28	− 0.29	− 0.31
Pay attention (in school)	− 0.26	− 0.27	− 0.16	− 0.32	− 0.30	− **0.43**	− 0.25	− 0.21	− 0.25
PedsQL^2^									
Physical	− 0.21	− 0.28	− **0.33**	− **0.29**	− 0.24	− 0.37	− 0.31	− **0.31**	− **0.36**
Psychosocial	− 0.31	− 0.40	− 0.33	− 0.40	− 0.36	− 0.44	− 0.39	− 0.40	− 0.35
Emotional	− **0.35**	− **0.43**	− 0.32	− **0.41**	− **0.36**	− 0.40	− **0.40**	− 0.38	− 0.30
Social	− 0.14	− 0.21	− 0.24	− 0.20	− 0.22	− 0.17	− 0.16	− 0.24	− **0.30**
School	− 0.24	− 0.33	− 0.28	− 0.35	− 0.27	− **0.48**	− 0.37	− 0.36	− 0.30

^1^KIDSCEEN-10 was completed by children in grades 2–9, n = 422

^2^PedsQL was completed by children in grades 1–9, n = 471

^3^Not statistically significant. All remaining Spearman’s correlations were statistically significant (*p* value < 0.05)

Correlations between conceptually alike dimensions are presented in bold

### Known-groups validity

Table 8 shows that CHU9D utility scores mostly differed as expected when comparing children with varying characteristics. As compared to their counterparts, higher scores were found among boys, younger children, those living with both parents, those reporting better general health and those without a long-term illness or disability (child and adult algorithms, both). However, there were no utility score differences depending on parental country of birth or family affluence. To further investigate the lack of statistical differences in utility scores by family affluence, we conducted several other analyses, in which, we stratified FAS-scores into 2, 4 and 5 categories with different set and relative cut-offs, tested with and without imputation for the 37 cases with missing FAS scores. All analyses confirmed the initial results (data not shown).

**Table 8:** Comparison of CHU9D utility scores between children with different sociodemographic and health characteristics (n = 473)

	CHU9D (Child algorithm)			CHU9D (Adult algorithm)		*p* value
	Mean (SD)	Median (IQR)	*p* value^1^	Mean (SD)	Median (IQR)	
Sex						
Girls	0.69 (0.22)	0.72 (0.52–0.89)	> 0.001	0.83 (0.11)	0.84 (0.75–0.93)	> 0.001
Boys	0.78 (0.18)	0.84 (0.68–0.91)		0.88 (0.09)	0.90 (0.82–0.95)	
School stages						
Grades 1–3	0.80 (0.18)	0.86 (0.70–0.95)	0.0001	0.88 (0.09)	0.91 (0.83–0.95)	0.0001
Grades 4–6	0.75 (0.20)	0.79 (0.62–0.89)		0.86 (0.10)	0.88 (0.79–0.93)	
Grades 7–9	0.65 (0.23)	0.66 (0.48–0.85)		0.81 (0.11)	0.82 (0.75–0.90)	
Parents’ country of birth						
None Sweden	0.76 (0.21)	0.80 (0.60–0.97)	0.322	0.87 (0.11)	0.90 (0.78–0.96)	0.303
One Sweden	0.72 (0.18)	0.70 (0.59–0.87)		0.85 (0.09)	0.85 (0.79–0.92)	
Both Sweden	0.73 (0.21)	0.79 (0.59–0.91)		0.85 (0.11)	0.87 (0.78–0.93)	
Custody arrangement						
Not living with both parents	0.69 (0.21)	0.71 (0.53–0.86)	0.018	0.83 (0.11)	0.83 (0.76–0.92)	0.025
Living with both parents	0.74 (0.21)	0.80 (0.59–0.91)		0.86 (0.11)	0.88 (0.78–0.93)	
General health						
Not good	0.48 (0.19)	0.47 (0.33–0.60)	0.0001	0.72 (0.10)	0.72 (0.65–0.78)	0.0001
Good	0.64 (0.19)	0.62 (0.48–0.79)		0.80 (0.10)	0.79 (0.74–0.88)	
Very good	0.81 (0.16)	0.86 (0.70–0.96)		0.89 (0.08)	0.91 (0.84–0.95)	
Long-term illness/disability						
Yes	0.71 (0.23)	0.78 (0.54–0.89)	0.008	0.84 (0.12)	0.87 (0.77–0.93)	0.048
No	0.77 (0.19)	0.80 (0.63–0.94)		0.87 (0.09)	0.88 (0.80–0.95)	
Family affluence						
Low (< 25percentile)	0.75 (0.19)	0.81 (0.60–0.92)	0.732	0.87 (0.10)	0.88 (0.78–0.95)	0.438
Middle (25–75percentile)	0.74 (0.20)	0.79 (0.60–0.89)		0.85 (0.10)	0.87 (0.79–0.93)	
High (> 75percentile)	0.71 (0.24)	0.79 (0.52–0.94)		0.84 (0.12)	0.87 (0.76–0.93)	

*IQR* inter quartile range, *SD* standard deviation

^1^*p* values estimated by use of Kruskal–Wallis equality-of-populations rank test and Mann–Whitney U (Wilcoxon rank sum)

The sample size only allowed grade-stratified analyses by sex and long-term illness/disability. In grades 4–6 and 7–9, these analyses showed similar pattern as those reported above, but for long-term illnesses, only when applying the child algorithm. In grades 1–3, no such differences were seen.

## Discussion

This study investigated the feasibility, reliability and construct validity of the Swedish CHU9D among school children attending grades 1–9 in Sweden (ages 7–15), and separately for children in grades 1–3, 4–6 and 7–9 (ages 7–9, 10–12 and 13–15 years).

Very few missing values, minimal ceiling, and no floor effects support the general feasibility of the Swedish CHU9D. However, ceiling effects were relatively high for several of the underlying CHU9D dimensions. This is not surprising given that many children are expected to be at good health in general populations. Similar results have been shown when using the English, Danish and Chinese CHU9D in general populations of school-aged children [13, 14, 31, 33, 36]. Notable though, across these studies, as well as in the current study, floor effects in the CHU9D dimensions are mainly below 5% and ceiling effects below 85%. This indicates that the CHU9D is capable of detecting improvement in general population studies of school-aged children, in Sweden and elsewhere.

The reliability of the Swedish CHU9D was supported by strong test–retest correlations and agreements, along with established internal consistency. Again, this is in line with the result of studies using the English, Danish and Chinese CHU9D in their cultural contexts [33, 36, 37]. Notably though, for most of the individual CHU9D dimension scores, we only found moderate or close to moderate test–retest correlations and agreements. Similar findings were reported in a UK study of 6–7-year-olds using a shorter test–retest timeframe (morning-to-afternoon) [31] and a Chinese two-week, test–retest study of 8–17-year-olds [36]. Thus, across language versions and cultural contexts, the CHU9D dimensions show signs of some inconsistency over time. This may be due to the shifting nature of the concepts studied, in combination with the short reference time (today). Furber et al. [37], reports that one third of the children do not consider the day of the study a typical day in terms of the assessed CU9D concepts, indicating that these concepts fluctuate day by day. Further research should investigate how this potential shortcoming influences the instruments sensitivity to change over time (responsiveness).

The current study confirms the proposed one-factor structure of the CHU9D. In the absence of a gold standard for HRQoL measurement, it is not possible to prove conclusively that this factor measures HRQoL. However, we found a strong correlation between the total scale score of the CHU9D and two HRQoL instruments with demonstrated reliability and validity (KIDSCREEN-10 and PedsQL). Furthermore, the strongest correlations were seen between the CHU9D dimensions and the conceptually overlapping KIDSCREEN-10 and PedsQL dimensions, while correlations were weaker for non-overlapping concepts. Other studies have shown similar results when comparing the original English CHU9D to KIDSCREEN-10 and PedsQL [14, 38] or the Danish and Chinese CHU9D to the PedsQL [33, 36]. Taken together, these results indicate that the CHU9D may be used as a measure of HRQoL.

Although we and other researchers [14, 33, 38] find correlations to be strongest between the CHU9D and alike KIDSCREEN-10 and PedsQL dimension, these correlations are merely moderate. This is not surprising, given that the questions and response alternatives are somewhat differently phrased in the three instruments. Also, the recall period is “today” in the CHU9D, while KIDSCREEN-10 and PedsQL have recall-times of one week and one month, respectively.

Consistent with studies from other countries, [12, 31, 33, 36] we confirmed that the Swedish CHU9D is able to detect anticipated HRQoL differences depending on health outcomes and sociodemographic characteristic such as sex, age and custody arrangement. We did however not replicate previously shown differences by socioeconomic status (SES) [13, 14] and ethnic origin [32]. This may be attributed to the fact that we required children to be fluent Swedish speakers, leading to the exclusion of families who are less rooted in the Swedish society and thereby potentially to less diversity by ethnicity and affluence. Thus, our results overall support the known-group validity of the Swedish CHU9D, but additional studies are required to confirm the ability to discriminate between children with different SES and ethnic origin.

The CHU9D was initially developed for ages 7–11 years [8]. Our study supports that the instrument is acceptable for use among children up to 15 years of age. Furthermore, in each of the three school stages studied, we found acceptable floor and ceiling effects, strongly correlated test–retest CH9D utility scores, an internal consistency allowing for group comparisons, and moderate to strong correlations between the CHU9D scale and two established HRQoL scales. These findings suggest that the CHU9D is feasible, reliable and valid for use, not only in wide age-ranges, but also in narrower strata comprising only children aged 7– 9 years, 10–12 year, or 13–15 years.

Our results confirm earlier findings showing that CHU9D utility scores are higher when applying the adult as compared to the child algorithm [9, 37, 39]. This may be attributed to the disparities in valuation methods used to assess utility weights in child (best–worst) and adult populations (standard gamble) [39]. It may also be explained by adults and children giving different values to CHU9D generated health states, i.e. that adults, as compared to children, generally place less weight on mental health impairment (*sadness, worries, being annoyed*) and impairment in daily functioning (*schoolwork, daily routines, activities*) but comparably higher weight on health states dominated by physical impairment (*pain, tiredness, sleep problems*) [6]. Thus, the CHU9D adult algorithm may not accurately reflect children’s preferences.

Notably, we found that the algorithm used influenced the size of between-groups differences. Comparing for instance those with a “*not* good” and a “*very* good” general health, we found utility scores difference to be twice as high when applying the child algorithm as compared with the adult algorithm (mean-score difference: 0.33 vs. 0.17). Such differences suggest that the choice of algorithm may have the capacity to influence interpretations of future economic evaluations of pediatric health interventions, highlighting the importance of this choice. We acknowledge some limitations. Although including only fluently Swedish speaking children diminishes biases due to language barriers, which is a strength, it may also have biased the discriminative analysis regarding SES and ethnic origin. Also, given that the study was based on a convenience sample, it cannot provide normative information about HRQoL levels. Another limitation is that this general population study with self- and parent-reported health, could not evaluate the applicability of the CHU9D in clinical population. Likewise, the study design did not allow evaluations of responsiveness to change in health status over time. In addition, the long-term illness/disability measure was based on self-report by children or parents, and were not confirmed via medical records.

## Conclusions

This study provides support that the Swedish CHU9D is a feasible, reliable and valid measure of HRQoL that holds psychometric qualities comparable to those of the original English CHU9D. The study furthermore, suggests that the CHU9D is appropriate for use among 7–15-year-old children in the general population, as well as among subgroups aged 7– 9, 10–12 and 13–15 years. To provide further support for the CHU9D as a useful health outcome measure in health economic evaluations, future studies should investigate the performance of the CHU9D in clinical samples. Longitudinal studies are also needed to test the instruments sensitivity to change.

## Data Availability

Not applicable.

## References

[1] Drummond MF, Sculpher MJ, Torrance GW, O'Brien BJ, Stoddart GL (2005). Methods for the economic evaluation of health care programmes.

[2] Chen G, Ratcliffe J (2015). A review of the development and application of generic multi-attribute utility instruments for paediatric populations. Pharmacoeconomics.

[3] Kwon J, Kim SW, Ungar WJ, Tsiplova K, Madan J, Petrou S (2018). A systematic review and meta-analysis of childhood health utilities. Med Decis Mak.

[4] Thorrington D, Eames K (2015). Measuring health utilities in children and adolescents: a systematic review of the literature. PLoS ONE.

[5] Eiser C, Morse R (2001). Quality-of-life measures in chronic diseases of childhood. Health Technol Assess.

[6] Ratcliffe J, Huynh E, Stevens K, Brazier J, Sawyer M, Flynn T (2016). Nothing about us without us? A comparison of adolescent and adult health-state values for the child health utility-9D using profile case best-worst scaling. Health Econ.

[7] Stevens K (2009). Developing a descriptive system for a new preference-based measure of health-related quality of life for children. Qual Life Res.

[8] Stevens K (2011). Assessing the performance of a new generic measure of health-related quality of life for children and refining it for use in health state valuation. Appl Health Econ Health Policy.

[9] Stevens KJ (2010). Working with children to develop dimensions for a preference-based, generic, pediatric, health-related quality-of-life measure. Qual Health Res.

[10] Stevens K (2012). Valuation of the child health utility 9D index. Pharmacoeconomics.

[11] Ratcliffe J, Huynh E, Chen G, Stevens K, Swait J, Brazier J, Sawyer M, Roberts R, Flynn T (2016). Valuing the child health utility 9D: using profile case best worst scaling methods to develop a new adolescent specific scoring algorithm. Soc Sci Med.

[12] Chen G, Flynn T, Stevens K, Brazier J, Huynh E, Sawyer M, Roberts R, Ratcliffe J (2015). Assessing the health-related quality of life of australian adolescents: an empirical comparison of the child health utility 9D and EQ-5D-Y instruments. Value Health.

[13] Ratcliffe J, Stevens K, Flynn T, Brazier J, Sawyer M (2012). An assessment of the construct validity of the CHU9D in the Australian adolescent general population. Qual Life Res.

[14] Stevens K, Ratcliffe J (2012). Measuring and valuing health benefits for economic evaluation in adolescence: an assessment of the practicality and validity of the child health utility 9D in the Australian adolescent population. Value Health.

[15] University of Sheffield. School of Health and Related Research. Measuring and Valuing Health. https://www.sheffield.ac.uk/scharr/research/themes/valuing-health#CHU-9D%20(Paediatric%20Quality%20of%20Life. Accessed 01 Sept 2020.

[16] WHO QoLAG. What quality of life?/The WHOQOL Group. World Health Forum. 1996;17(4):354–6. 9060228

[17] Lohr KN (2002). Assessing health status and quality-of-life instruments: attributes and review criteria. Qual Life Res.

[18] Ravens-Sieberer U, Erhart M, Rajmil L, Herdman M, Auquier P, Bruil J, Power M, Duer W, Abel T, Czemy L (2010). Reliability, construct and criterion validity of the KIDSCREEN-10 score: a short measure for children and adolescents’ well-being and health-related quality of life. Qual Life Res.

[19] Ravens-Sieberer U, Gosch A, Erhart M, Rueden U, Nickel J, Kurth B-M, Duer W, Fuerth K, Czemy L, Auquier P. The KIDSCREEN Questionnaires—quality of life questionnaires for children and adolescents—handbook. Lengerich: Papst Science Publisher; 2006.

[20] Varni JW, Burwinkle TM, Seid M (2006). The PedsQL TM 4.0 as a school population health measure: feasibility, reliability, and validity. Qual Life Res.

[21] Varni JW, Seid M, Kurtin PS. PedsQL™ 4.0: Reliability and validity of the pediatric quality of life inventory™ version 4.0 generic core scales in healthy and patient populations. Med Care. 2001;800–12. 10.1097/00005650-200108000-00006 11468499

[22] Stevens K. The child health utility 9D (CHU9D)—a new paediatric preference based measure of health related quality of life. In: PRO Newsletter. vol. 43; 2010.

[23] Petersen S, Hägglöf B, Stenlund H, Bergström E (2009). Psychometric properties of the Swedish PedsQL, Pediatric Quality of Life Inventory 4.0 generic core scales. Acta Paediatr..

[24] Torsheim T, Cavallo F, Levin KA, Schnohr C, Mazur J, Niclasen B, Currie C, Group FDS (2016). Psychometric validation of the revised family affluence scale: a latent variable approach. Child Indic Res..

[25] Aaronson N, Alonso J, Burnam A, Lohr KN, Patrick DL, Perrin E, Stein RE (2002). Assessing health status and quality-of-life instruments: attributes and review criteria. Qual Life Res.

[26] Evans JD (1996). Straightforward statistics for the behavioral sciences.

[27] Landis JR, Koch GG. The measurement of observer agreement for categorical data. Biometrics. 1977;159–74. 843571

[28] Marsh HW, Hau K-T, Wen Z (2004). In search of golden rules: Comment on hypothesis-testing approaches to setting cutoff values for fit indexes and dangers in overgeneralizing Hu and Bentler's (1999) findings. Struct Equ Model.

[29] Mw NE (1993). Cudeck R: alternative ways of assessing model fit. Test Struct Equ Models.

[30] Hu LT, Bentler PM. Fit indices in covariance structure modeling: sensitivity to underparameterized model misspecification. Psychol Methods. 1998;3(4):424.

[31] Canaway AG, Frew EJ (2013). Measuring preference-based quality of life in children aged 6–7 years: a comparison of the performance of the CHU-9D and EQ-5D-Y–the WAVES pilot study. Qual Life Res.

[32] Frew EJ, Pallan M, Lancashire E, Hemming K, Adab P (2015). Is utility-based quality of life associated with overweight in children? Evidence from the UK WAVES randomised controlled study. BMC Pediatr.

[33] Petersen KD, Ratcliffe J, Chen G, Serles D, Frøsig CS, Olesen AV (2019). The construct validity of the Child Health Utility 9D-DK instrument. Health Qual Life Outcomes.

[34] Terwee CB, Bot SD, de Boer MR, van der Windt DA, Knol DL, Dekker J, Bouter LM, de Vet HC (2007). Quality criteria were proposed for measurement properties of health status questionnaires. J Clin Epidemiol.

[35] Kline P. The handbook of psychological testing. 1993.

[36] Yang P, Chen G, Wang P, Zhang K, Deng F, Yang H, Zhuang G (2018). Psychometric evaluation of the Chinese version of the Child Health Utility 9D (CHU9D-CHN): a school-based study in China. Qual Life Res.

[37] Furber G, Segal L (2015). The validity of the Child Health Utility instrument (CHU9D) as a routine outcome measure for use in child and adolescent mental health services. Health Qual Life Outcomes.

[38] Petersen KD, Chen G, Mpundu-Kaambwa C, Stevens K, Brazier J, Ratcliffe J (2018). Measuring health-related quality of life in adolescent populations: an empirical comparison of the CHU9D and the PedsQL TM 4.0 short form 15. Patient-Patient-Center Outcomes Res..

[39] Ratcliffe J, Stevens K, Flynn T, Brazier J, Sawyer MG (2012). Whose values in health? An empirical comparison of the application of adolescent and adult values for the CHU-9D and AQOL-6D in the Australian adolescent general population. Value Health.

